# A Polymorphism in *IL28B* Distinguishes Exposed, Uninfected Individuals From Spontaneous Resolvers of HCV Infection

**DOI:** 10.1053/j.gastro.2011.04.005

**Published:** 2011-07

**Authors:** Susanne Knapp, Usama Warshow, K.M. Alexander Ho, Doha Hegazy, Ann–Margaret Little, Andrew Fowell, Graeme Alexander, Mark Thursz, Matthew Cramp, Salim I. Khakoo

**Affiliations:** ⁎ Department of Hepatology, Division of Medicine, Imperial College London, United Kingdom; ‡ Hepatology Research Group, Peninsula Medical School and South West Liver Unit, Derriford Hospital, Plymouth, United Kingdom; § Laboratory of Histocompatibility and Immunogenetics, Gartnavel General Hospital, Glasgow, United Kingdom; ∥ Department of Hepatology, Southampton General Hospital, Southampton, United Kingdom; ¶ Department of Medicine, University of Cambridge, Cambridge, United Kingdom

**Keywords:** Killer Cell Immunoglobulin-Like Receptor, Genetics, Liver Disease, Protective Mechanisms, EU, exposed but uninfected, HCV, hepatitis C virus, Hencore, Hepatitis C European Network for Cooperative Research collaboration, HLA, human leukocyte antigen, HLA-C1, group 1 HLA-C allotype, IDU, injection drug users, IFN, interferon, KIR, killer cell immunoglobulin-like receptor, NK, natural killer cells, SNP, single nucleotide polymorphism, SR, spontaneous resolvers

## Abstract

**Background & Aims:**

Polymorphisms in the interleukin-28B (*IL28B*) gene are associated with outcomes from infection with hepatitis C virus (HCV). However, the role of these polymorphisms in protecting injection drug users who are at high risk for HCV infection but do not have detectable antibodies against HCV or HCV RNA (exposed uninfected) has not been demonstrated. We investigated whether these individuals have the *IL28B* genotype *rs12979860-CC*, which protects some individuals against HCV infection.

**Methods:**

Seventy-four exposed uninfected individuals, 89 spontaneous resolvers, and 234 chronically infected individuals were genotyped to determine single nucleotide polymorphisms at *IL28B.rs12979860*.

**Results:**

Exposed, uninfected individuals had a significantly lower frequency of the protective genotype *(rs12979860-CC*) than anti-HCV-positive spontaneous resolvers (41.9% vs 69.7%, respectively; *P* = .0005; odds ratio [OR], 0.31; 95% confidence interval [CI]: 0.16–0.60) but a similar frequency to patients who were chronically infected (41.9% vs 43.6%, respectively; *P* = ns). However, exposed, uninfected individuals had a significantly higher frequency of homozygosity for killer cell immunoglobulin-like receptor 2DL3:group 1 HLA-C (*KIR2DL3:HLA-C1*) than those with chronic infection (31.1% vs 13.3%, respectively; *P* = .0008; OR, 2.95; 95% CI: 1.59–5.49). For patients who spontaneously resolved infection, *IL28B* and *KIR:HLA* protected, independently, against chronic HCV infection, based on logistic regression and synergy analyses (synergy factor, 1.3; 95% CI: 0.37–4.75; *P* synergy = .6).

**Conclusions:**

*IL28B* and *KIR2DL3:HLA-C1* are independently associated with spontaneous resolution of viremia following HCV exposure. Resistance to HCV infection in exposed uninfected cases is associated with homozygosity for *KIR2DL3:HLA-C1* but not the single nucleotide polymorphism *IL28B.rs12979860*. Uninfected individuals are therefore a distinct population from patients who spontaneously resolve HCV infection. Distinct, nonsynergistic innate immune mechanisms can determine outcomes of HCV exposure.

Hepatitis C virus (HCV) is a common chronic viral infection with only a minority of individuals exposed to HCV infection being able to resolve infection spontaneously. Clearance of HCV is dependent on a successful immune response, which likely involves T cells, B cells, dendritic cells, and also natural killer cells (NK) cells.^1^ Consistent with a broad immune response being important, polymorphisms of both the innate and adaptive immune system are associated with spontaneous resolution of HCV infection.^2^

Recent work has highlighted that polymorphisms in the Interleukin-28B (*IL28B*) gene (interferon [IFN]-λ3) are strongly associated with both spontaneous resolution of HCV infection and also resolution of infection with pegylated interferon and ribavirin.^2–7^ Similarly, the killer cell immunoglobulin-like receptors (KIR) and their human leukocyte antigen class I ligands have also been implicated in spontaneous and treatment-induced resolution of HCV infection.^8–11^ In particular, KIR2DL3 and its ligands, the group 1 HLA-C allotypes (HLA-C1), are protective against chronic HCV infection and, hence, are beneficial factors in outcome following exposure to HCV.

A minority of long-term injection drug users (IDU) demonstrate apparent resistance to HCV infection and remain seronegative and aviremic despite likely repeated exposure to HCV through the sharing of drug injection equipment. These exposed but uninfected (EU) IDU cases have been shown to have detectable HCV-specific T-cell responses, indicating their exposure to HCV infection.^12,13^ They also have increased NK cell activity.^14^ Consistent with this, we have recently shown that, similar to conventional spontaneous resolvers (SR), the combination of *KIR2DL3* and *HLA-C1* is also over-represented in the exposed seronegative aviremic population.^10^ Additionally, both groups of protected individuals have an increased frequency of a functional interleukin-12 (*IL-12*) polymorphism as compared with chronically infected individuals.^15,16^

To date, the protective effect of *IL28B* in this subgroup of individuals has not been investigated. Furthermore, it is not well understood whether protective polymorphisms in the immune system work together to increase protection against chronic HCV infection or whether these components of the innate immune system act independently. The aim of this study was therefore to determine whether the EU population have a protective *IL28B* genotype and to determine how protective *IL28B* and *KIR:HLA-C* polymorphisms may interact to influence the outcome of HCV infection in untreated individuals.

## Patients and Methods

### Patients

Three hundred ninety-seven patients (74 exposed uninfected, 89 SR, and 234 chronically infected patients) were studied for the distribution of the *IL28B.rs12979860* single nucleotide polymorphism (SNP), *KIR2DL2/3 and HLA-C* genotypes. All patients gave informed consent with approval by the relevant ethics committees as previously described.^8,10,17^ Patients were excluded if they were human immunodeficiency virus positive or hepatitis B virus surface antigen positive. The patients are classified into the following 3 cohorts: (1) exposed uninfected (EU) cohort, (2) spontaneous resolving (SR), and (3) chronically infected individuals.

#### Exposed uninfected cohort

Seventy-four individuals were recruited from Dartmoor Prison, needle exchanges, community drug services, and hostels in Plymouth, United Kingdom. All these individuals were of Caucasian ethnicity. They had an extensive history of past or present injection drug use. This group was defined as being both HCV antibody (third generation enzyme linked immunosorbent assay, Abbott IMx, Abbott Diagnostics, Maidenhead, Berkshire, United Kingdom) and HCV RNA (Amplicor, Roche Diagnostics, Pleasanton, CA) negative on at least 2 occasions, 3–6 months apart with subsequent testing on an approximate 6 monthly basis to ensure that this profile remained unchanged. Forty-two of these cases had been genotyped previously for KIR2DL2/3 and HLA-C.^10^ Detailed information about drug injecting behavior was ascertained by means of a structured questionnaire, and the median duration of intravenous drug use was 8.62 ± 6.05 years (range, 0.3–24) with a median number of injections of 4927 (range, 36–41,620).^10^ Their median age was 28 years, and 64 (79%) were male.

**SRs.** Individuals were classified in this group if they had detectable anti-HCV by second-generation enzyme-linked immunosorbent assay (Abbott IMx; Abbott Diagnostics, Maidenhead, Berkshire, United Kingdom) and no detectable HCV viremia by Quantiplex HCV RNA 2.0 assay (Chiron, Emeryville, CA) or HCV COBAS Amplicor system (Roche Diagnostics, Pleasanton, CA) on at least 2 occasions 6 months apart. They were recruited between 1995 and 1998 as part of the Hepatitis C European Network for Cooperative Research (Hencore) collaboration^17,18^ and between 1999 and 2005 from Addenbrookes Hospital, Cambridge, United Kingdom, and Southampton General Hospital, United Kingdom.^8^ Eighty-seven (98%) were Caucasian, 59 (66%) were male, and their median age was 36 years. Forty-four had been genotyped previously for *KIR2DL2/3* and *HLA-C.*^8^

#### Chronically infected individuals

These individual were all persistently anti-HCV and HCV RNA positive, by second-generation enzyme-linked immunosorbent assay (Abbott IMx) and HCV COBAS Amplicor system (Roche Diagnostics, Pleasanton, CA), respectively. They were recruited from the general hepatology clinic at Southampton General Hospital, United Kingdom, between 2003 and 2007. Two hundred seventeen (93%) were of Caucasian origin, with a median age of 45 years, and 138 (59%) were male.^10^ All had been genotyped previously for *KIR2DL2/3* and *HLA-C*.^8^

### Genotyping

Genomic DNA was extracted from peripheral blood lymphocytes using a salt precipitation method,^17^ Nucleon DNA extraction kit (Tepnel Lifesciences, Manchester, United Kingdom) or the QIAamp blood kit (Qiagen, Crawley, United Kingdom). All samples were typed for the *rs12979860* SNP using a real-time polymerase chain technique incorporating Sybr Green (Qiagen QuantiTect SYBR; Qiagen). The primers used were as follows: 5′-GCTTATCGCATACGGCTAGGC-3′ (forward common), 5′-GCAATTCAACCCTGGTTCG-3′ (C- allele specific reverse) and 5′-GCAATTCAACCCTGGTTCA-3′ (T-allele specific reverse). Reactions were performed on a 5700 Perkin Elmer (Cambridge, United Kingdom) machine using 96-well plates and 10–100 ng genomic DNA with 0.5 μmol/L of each primer in a reaction mix of total volume 20 μL. The thermal cycling protocol consisted of an initial denaturation step of 95°C for 10 minutes, followed by 40 two-step amplification cycles of 95°C for 20 seconds and 58°C for 20 seconds.

*KIR2DL2/3* genotyping was performed on the Hencore cohort and the 32 additional exposed uninfected individuals by polymerase chain reaction using sequence specific primers as previously described.^19^ HLA typing was performed on the Hencore and EU cohorts as described elsewhere.^20^ *HLA* types that were not resolved by sequencing or that gave unusual results were also tested by sequence-specific oligonucleotide probe typing using commercial kits (RELI SSO; Dynal, Wirral, United Kingdom). Other cohorts had previously been typed for *KIR2DL2/3 and HLA-C.*^8,10^

### Statistical Analysis

GraphPad Prism 5 software (GraphPad, Inc, La Jolla, CA) was used to calculate 2-tailed *P* values and odds ratios (OR) from 2 × 2 contingency tables by Fisher exact test. Logistic regression analysis was performed using SPSS statistical software version 17 (SPSS, Inc, Chicago, IL) with the ENTER function. Synergy between *IL28B* and *KIR:HLA* was calculated using the method of Cortina-Borja et al.^21^

## Results

### IL28B Polymorphism Distinguishes Exposed Uninfected Individuals From Anti-HCV-Positive Spontaneous Resolvers

The frequency of the protective *CC* genotype at the SNP *rs12979860-CC* in the 74 EU individuals was significantly lower than in the 89 SR (41.9% vs 69.7%, respectively, *P* = .0005; OR, 0.31; 95% confidence interval [CI]: 0.16–0.60) but was similar to that found in the 234 individuals with chronic HCV infection (41.9% vs 43.6%, respectively) (Table 1). Consistent with previous work, the frequency of the *IL28B.rs12979860-CC* genotype was significantly higher in the spontaneous resolving population compared with those with chronic infection (69.7% vs 43.6%, respectively, *P* < .0001; OR, 2.97, 95% CI: 1.76–5.00). We also found that *CT* heterozygosity was more prevalent in the EU as compared with the SR population (43.2% vs 24.7%, respectively, *P* = .019; OR, 2.32, 95% CI: 1.19–4.52), and this genotype was lower in the SR population as compared with the chronically infected individuals (24.7% vs 48.7%, respectively, *P* < .0001; OR, 0.35, 95% CI: 0.20–0.60). Additionally, we found that there was a trend toward an increase in *TT* homozygosity in the EU population as compared with both SR (14.9% vs 5.6%, respectively, *P* = .06; OR, 2.93, 95% CI: 0.97–8.87) and also chronically infected individuals (14.9% vs 7.7%, respectively, *P* = .07; OR, 2.09, 95% CI: 0.94–4.67). This is despite the overall *T* allele frequency being similar between EU and chronically infected individuals (36.5% vs 32.1%, respectively) (Supplementary Tables 1 and 2). These observations remained similar if only Caucasian individuals were considered (Supplementary Table 3). Thus, the *rs12979860* polymorphism distinguishes the EU population from those that spontaneously resolve HCV infection.

### IL28B and KIR:HLA Define Distinct Populations of HCV Protected Individuals

Although *IL28B.rs12979860-CC* was not associated with protection in the EU cohort, these individuals are genetically distinct from those with chronic HCV because homozygosity for *KIR2DL3*:*HLA-C1* is over-represented in this population as compared with those with chronic HCV (31.1% vs 13.3%, respectively, *P* = .0008; OR, 2.95, 95% CI: 1.59–5.49) (Supplementary Table 4). *KIR2DL3:HLA-C1* was found at a similar frequency to the anti-HCV-positive SR population (31.1% vs 29.2%, respectively, *P* = ns), as we have previously shown in a subgroup of these individuals.^10^ We therefore hypothesized that *KIR* and *IL28B* genes might define distinct groups of individuals who are protected against chronic HCV infection using different genetic pathways. To study the interrelationship of these genes on the outcome of hepatitis C, we compared the frequency of *IL28B.rs12979860-CC* in individuals with and without the protective *KIR2DL3:HLA-C1* homozygous genotype from all 3 cohorts (EU, SR, and chronic).

In individuals who had spontaneously resolved infection and were not *KIR2DL3:HLA-C1* homozygous, the frequency of the *rs12979860-CC* genotype was significantly higher compared with chronically infected individuals (68.3% [SR] vs 41.9% [chronic], *P* = .0003; OR, 2.98, 95% CI: 1.64–5.43, Table 2). The effect was similar in individuals who were *KIR2DL3:HLA-C1* homozygous, but this did not reach statistical significance (73.1% vs 54.8%, respectively, *P* = .18; OR, 2.23, 95% CI: 0.73–6.84), most likely because of the small sample size. Likewise, the protective effect of *KIR2DL3:HLA-C1* homozygosity was similar in individuals with the *rs12979860-CC* genotype (30.6% [SR] vs 16.7% [chronic], *P* = .051; OR, 2.21, 95% CI: 1.04–4.68) and also without the *rs12979860-CC* genotype (25.9% SR vs 10.6% chronic, *P* = .055; OR, 2.95, 95% CI: 1.06–8.21). Similarly, we found an under-representation of *rs12979860-CC* in EU as compared with SR in both the *KIR2DL3:HLA-C1* homozygous and nonhomozygous subgroups (*P* = .046; OR, 0.28, 95% CI: 0.09–0.94 and *P* = .0046; OR, 0.33, 95% CI: 0.15–0.70, respectively, Table 2).

In univariate analysis, the frequency of the combination of *rs12979860-CC* and *KIR2DL3:HLA-C1* homozygosity in the SR group was 21% as compared with only 7.3% in the chronically infected group (*P* = .0007; OR, 3.47, 95% CI: 1.71–7.03). However, it is not clear whether these 2 protective genetic factors are acting synergistically or independently. To determine this, we performed multivariate logistic regression analysis using 3 variables: *rs12979860-CC+KIR2DL3:HLA-C1* homozygosity; *rs12979860-CC* with or without *KIR2DL3:HLA-C1* homozygosity; and *KIR2DL3:HLA-C1* homozygosity with or without *rs12979860-CC*. This analysis tests whether the combination of the 2 factors provides additional benefit above that due to each factor individually. This demonstrated that the *rs12979860-CC* genotype and *KIR2DL3:HLA-C1* homozygosity are protective in isolation (*P* < .001 and *P* = .04, respectively), and having both *rs12979860-CC* and *KIR2DL3:HLA-C1* homozygosity together does not confer any additional protection (*P* > .1) (Table 3). Additionally, we applied a recently described test to evaluate the synergistic effects of genetic factors.^21^ This method is based on logistic regression analysis and compares the ORs of protection among the different groups. It determines whether the observed OR for 2 factors considered in combination is greater than that of having both protective factors assuming independent effects of each factor. In this case, 4 groupings were tested: (1) *rs12979860-CC* positive, not *KIR2DL3:HLA-C1* homozygous; (2) *KIR2DL3:HLA-C1* homozygous, *rs12979860-CC* negative; (3) *rs12979860*-CC positive and *KIR2DL3:HLA-C1* homozygous; and (4) neither *rs12979860-CC* positive nor *KIR2DL3:HLA-C1* homozygous. Using this test, we confirmed the absence of synergy between the 2 protective factors in the SR population (synergy factor = 1.3 [95% CI: 0.37–4.75], *P*_synergy_ = .6). Because the synergy factor can uncover unexpected synergies, we determined this statistic for the EU population in comparison with the chronically infected individuals. However, no synergy was found (synergy factor = 1.53 [95% CI: 0.44–5.37], *P*_synergy_ = .5). Thus, these polymorphisms of the innate immune system distinguish EU from both SR and chronically infected individuals and operate independently to protect individuals against chronic HCV infection.

## Discussion

HCV causes chronic infection in the majority of exposed individuals, thus protection from HCV infection is the exception rather than the norm. Individuals with beneficial immune responses have traditionally been identified as anti-HCV positive, HCV RNA negative. More recently, individuals who remain seronegative and aviremic despite high-risk behavior have also been shown to be relatively protected against chronic infection. These individuals have detectable T-cell responses,^12,13,22^ a favorable *KIR2DL3:HLA-C* genotype,^10^ and also a protective *IL12* genotype.^15,16^ In this respect, they are indistinguishable from conventional SR. However, our data show that protection in this subgroup of individuals is not associated with the *IL28B.rs12979860-CC* genotype, which marks them as distinct from SR. Indeed, they are the first subgroup of individuals identified who have a favorable outcome following HCV exposure who do not have an over-representation of this genotype. This is unlikely to represent a bias related to the ethnicity of our population because all EU individuals were Caucasian, and the frequency of the *IL28B.rs12979860-C* allele (63.5%, Supplementary Table 2) is comparable with that of 67.4% reported by Thomas et al in Americans of European extraction and is also similar to the frequency found in other European populations.^6^ Therefore, it seems that *IL28B* distinguishes the population of SR from other healthy and HCV exposed populations.

Overall, given our understanding of the protective nature of the *rs12979860-CC* genotype, it may be that this genotype fails to deliver protection against acute HCV infection. One alternative explanation could be that the *rs12979860TT* genotype is protective against acute HCV infection. Potentially, this genotype could be associated with a weaker antibody response and a bias toward both innate and adaptive cell mediated immunity. Interestingly, the *rs12979860-TT* genotype was over-represented in our EU cohort as compared with both SR and chronically infected individuals, consistent with a role in skewing the immune response away from antibody production. This difference is unlikely to be related to a population bias because the trend was present when Caucasian individuals alone were considered, and, also, the overall *T* allele frequency was similar between EU and chronically infected individuals.

Within the spontaneously resolving group are 2 distinct populations: those resolving HCV via an *IL28B*-associated mechanism and those with a protective *KIR:HLA* combination. We found that the combination of *KIR2DL3:HLA-C1* and *IL28B.rs12979860-CC* homozygosity did not provide any additional protection above that due to each genetic factor in isolation as determined both by logistic regression and calculation of a synergy factor. This indicates that they function as independent genetic protective factors and do not have a synergistic interaction. The calculation of a synergy factor allows separation of a true synergistic interaction from an apparent one, that is, one that is due to the expected increase in OR caused by combining 2 protective factors.^21^ This analysis also complements that performed by logistic regression, which demonstrated that the combination of the 2 protective factors had no advantage over that due to each factor in isolation. Additionally, the synergy factor is designed to be robust for small samples sizes, even when individual cells are zero.^21^ Thus, overall, the absence of a synergistic interaction between these factors is consistent with the observation that *KIR:HLA*, but not *IL28B*, is protective in the EU cohort.

Both *KIR2DL3:HLA-C1* and *IL28B* have predominantly innate immune functions. *KIR2DL3*-positive NK cells are activated in the acute phase of HCV infection, and we have shown that *KIR2DL3*-positive NK cells from individuals who resolve HCV have higher levels of degranulation than healthy controls, but those from individuals who become chronically infected do not.^23^ Thus, *KIR2DL3* protection operates at the level of the NK cell. At present, the mechanism of action of *IL28B* in resolving HCV infection, either spontaneously or with treatment, is not clear. Although, as a type III interferon that shares signaling pathways with type I interferons,^24^ it most likely protects via a direct mechanism on the hepatocyte, possibly inhibiting HCV replication like the related molecule IFN-λ1^25^ or rendering cells less susceptible to infection.^26^ Additionally, IFN-λ2 does not appear to directly affect NK cells.^27^ Therefore, there is a biologic rationale for the separation of these 2 genetic effects.

One model for resistance to, or resolution of, HCV infection is that possession of multiple independent protective factors may synergize to provide protection against chronic infection so that individuals with more protective factors have a greater chance of resolution of HCV infection. Our data do not support this hypothesis. Instead, we propose that *KIR:HLA* and *IL28B* define 2 genetically distinct subpopulations of individuals who are relatively protected against chronic HCV infection. Future genetic studies of resolution of HCV infection should stratify for these genotypes to take this heterogeneity into account.

## Figures and Tables

**Supplementary Table 1:** Frequency and Comparisons of the *IL28B.rs12979860-T* Alleles in 74 Exposed Uninfected, 89 Spontaneous Resolvers, and 234 Chronically Infected HCV Patients

*rs12979860-T*	2N (%)	*P* value (*P*_c_)	OR (95% CI)
EU vs SR	54 (36.5) vs 32 (18.0)	.0002 (0.0006)	2.62 (1.58−4.36)
EU vs chronic	54 (36.5) vs 150 (32.1)	>.1	1.22 (0.83−1.79)
SR vs chronic	32 (18.0) vs 150 (32.1)	.0004 (.0012)	0.46 (0.30−0.71)

NOTE. Two-tailed *P* values were calculated for 2 × 2 contingency tables using Fisher exact test, and the Bonferroni correction was applied (*P*_c_). 95% CI, 95% confidence interval; chronic, chronically infected HCV patients; 2N, number of alleles; OR, odds ratio.

**Supplementary Table 2:** Frequency and Comparisons of the *IL28B.rs12979860-C* Alleles in 74 Exposed Uninfected, 89 Spontaneous Resolvers, and 234 Chronically Infected HCV Patients

*rs12979860-C*	2N (%)	*P* value (*P*_c_)	OR (95% CI)
EU vs SR	94 (63.5) vs 146 (82.0)	.0002 (.0006)	0.38 (0.23−0.63)
EU vs chronic	94 (63.5) vs 318 (67.9)	>.1	0.82 (0.56−1.21)
SR vs chronic	146 (82.0) vs 318 (67.9)	.0004 (.0012)	2.15 (1.40−3.31)

NOTE. Two-tailed *P* values were calculated for 2 × 2 contingency tables using Fisher exact test, and the Bonferroni correction was applied (*P*_c_). 95% CI, 95% confidence interval; chronic, chronically infected HCV patients; OR, odds ratio.

**Supplementary Table 3:** Comparison of the *IL28B.rs12979860-CC, -CT, and -TT* Genotypes in Caucasian Individuals

Genotypes	EU, n (%)	SR, n (%)	Chr, n (%)	EU vs SR	EU vs Chr	SR vs Chr
CC	31 (41.9)	60 (69.0)	93 (42.9)	*P* = .0008 (*P*_c_ = .002) OR = 0.32 95% CI: 0.17−0.62	*P* > .1	*P* < .0001 (*P*_c_ = .0002) OR = 2.96 95% CI: 1.75−5.02
CT	32 (43.2)	22 (25.3)	106 (48.8)	*P* = .019 (*P*_c_ = .06) OR = 2.25 95% CI: 1.16−4.39	*P* > .1	*P* = .0002 (*P*_c_ = .0006) OR = 0.35 95% CI = 0.20−0.62
TT	11 (14.9)	5 (5.7)	18 (8.3)	*P* = .07 (*P*_c_ = .2) OR = 2.86 95% CI: 0.05−8.67	*P* > .1	*P* > .1

NOTE. Includes 74 exposed uninfected, 87 spontaneous resolvers, and 217 chronically infected Caucasian individuals. Two-tailed *P* values were calculated for 2 × 2 contingency tables using Fisher exact test, and the Bonferroni correction was applied (*P*_c_). 95% CI, 95% confidence interval; Chr, chronically infected HCV patients; 2N, number of alleles; OR, odds ratio.

**Supplementary Table 4:** Frequency of *KIR2DL3:HLA-C1* Homozygosity in the Whole Cohort

	*KIR2DL3:C1* homozygosity, n (%)	*P* value (*P*_c_)	OR (95% CI)
EU vs SR	23 (31.1) vs 26 (29.2)	.86	0.91 (0.47−1.79)
EU vs chronic	23 (31.1) vs 31 (13.3)	.0008 (.002)	2.95 (1.59−5.49)
SR vs chronic	26 (29.2) vs 31 (13.3)	.002 (.005)	2.70 (1.49−4.89)

NOTE. Two-tailed *P* values were calculated for 2 × 2 contingency tables using Fisher exact test, and the Bonferroni correction was applied (*P*_c_). 95% CI, 95% confidence interval; chronic, chronically infected HCV patients; OR, odds ratio.

**Table 1:** Frequency of the *IL28B.rs12979860-CC*, *CT*, *and TT* Genotype in 74 Exposed Uninfected, 89 Spontaneous Resolvers, and 234 Chronically Infected HCV Patients

*rs12979860* Genotype	EU, n (%)	SR, n (%)	Chr, n (%)	EU vs SR	EU vs Chr	SR vs Chr
CC	31 (41.9)	62 (69.7)	102 (43.6)	*P* = .0005 (*P*_c_ = .002) OR = 0.31 95% CI: 0.16−0.60	*P* > .1	*P* < .0001 (*P*_c_ = .0002) OR = 2.97 95% CI: 1.76−5.00
CT	32 (43.2)	22 (24.7)	114 (48.7)	*P* = .019 (*P*_c_ = .057) OR = 2.32 95% CI: 1.19−4.52	*P* > .1	*P* < .0001 (*P*_c_ = .0002) OR = 0.35 95% CI: 0.20−0.60
TT	11 (14.9)	5 (5.6)	18 (7.7)	*P* = .06 (*P*_c_ > .1) OR = 2.93 95% CI: 0.97−8.87	*P* = .07 (*P*_c_ > .1) OR = 2.09 95% CI: 0.94−4.67	*P* > .1

NOTE. Two-tailed *P* values were calculated for 2 × 2 contingency tables using Fisher exact test, and the Bonferroni correction was applied (*P*_c_). 95% CI, 95% confidence interval; Chr, chronically infected HCV patients; OR, odds ratio.

**Table 2:** Effect of *rs12979860-CC* Homozygosity in 89 Spontaneously Resolving, 234 Chronically Infected, and 74 Exposed Uninfected Individuals With and Without the *KIR2DL3:HLA-C1* Genotype

	Comparison (n)	*rs12979860-CC* positive, n (%)	*P* value	OR (95% CI)
*KIR2DL3:C1* homozygous	SR (26) vs Chr (31)	19 (73.1) vs 17 (54.8)	>.1	2.23 (0.73−6.84)
	EU (23) vs SR (26)	10 (43.5) vs 19 (73.1)	.046 (*P*_c_ > .1)	0.28 (0.09−0.94)
*Not KIR2DL3:C1* homozygous	SR (63) vs Chr (203)	43 (68.3) vs 85 (41.9)	.0003 (*P*_c_ = .0009)	2.98 (1.64−5.43)
	EU (51) vs SR (63)	21 (41.2) vs 43 (68.3)	.0046 (*P*_c_ = .013)	0.33 (0.15−0.70)

NOTE. Two-tailed *P* values were calculated for 2 × 2 contingency tables using Fisher exact test, and the Bonferroni correction was applied (*P*_c_). 95% CI, 95% confidence interval; Chr, chronically infected HCV patients; OR, odds ratio.

**Table 3:** Multivariate Logistic Regression Analysis of the Interaction Between Individual Protective Factors in 89 Spontaneous Resolvers and 234 Chronically Infected Individuals

	SR, n (%)	Chronic, n (%)	*P* value	OR	95% CI
*860-CC*	62 (69.7)	102 (43.6)	<.001	2.99	1.64−5.43
*2DL3:C1* homozygous	26 (29.2)	31 (13.2)	.04	2.94	1.06−8.20
*860CC+2DL3:C1* homozygous	19 (21.3)	17 (7.3)	>.1	0.75	0.21−2.67

NOTE. The 3 variables included in this analysis were *IL28B.rs12979860CC* (*860-CC*), *KIR2DL3:HLA-C* group 1 homozygosity (*2DL3-C1*), and *IL28B.rs12979860CC* in combination with *KIR2DL3:HLA-C group 1* homozygosity (*860-CC+2DL3-C1*). 95% CI, 95% confidence interval; Chronic, chronically infected HCV patients; OR, odds ratio.
